# Modification of navigated S2–alar iliac minimally invasive pelvic fixation to facilitate ease of rod passage

**DOI:** 10.3171/2026.4.FOCVID2648

**Published:** 2026-07-01

**Authors:** Ali Moghaddamjou, Nina Yoh, Nathan Winans, Andrew Chan, Praveen V. Mummaneni, Dean Chou

**Affiliations:** 1 Department of Neurological Surgery, Columbia University, NewYork-Presbyterian, Och Spine Hospital, New York, New York; and; 2 Department of Neurological Surgery, University of California, San Francisco, California

**Keywords:** pelvic fixation, S2-AI screw, minimally invasive surgery, navigation, operative technique

## Abstract

Rod alignment in minimally invasive spine (MIS) pelvic fixation using a traditional S2–alar iliac (S2-AI) starting point can be challenging, often requiring substantial rod contouring because of the medial to lateral angulation of the S2-AI tulip relative to the S1 screw. The authors describe a modified pelvic fixation strategy to optimize alignment between the pelvic screw and S1 screw through a more lateral entry point, near the sacroiliac joint. This adjustment facilitates smoother rod passage and easier set screw seating. Intraoperative navigation is critical in safely identifying the starting point and planning the construct alignment.

The video can be found here: https://stream.cadmore.media/r10.3171/2026.4.FOCVID2648

## Transcript

This video is on a modification of navigated S2-AI minimally invasive pelvic fixation to facilitate ease of rod passage.

### 0:28 Procedural Rationale.

The procedural rationale for this technique is that rod alignment with MIS pelvic screws can be difficult. Furthermore, rod passage can require significant contouring or significant reduction maneuvers, and rod passage may increase operative time and complexity of surgery. The benefits of this modification of the entry point is that direct alignment of the lumbosacral construct to the pelvic screws can be achieved, there’s easier rod passage and set screw seating, less rod contouring, and reduced operative time.

### 0:59 Limitations of Current Techniques.

For traditional iliac screws,^1^ they can be prominent over the posterior superior iliac spine and oftentimes require offset connectors due to difficult rod connection. For traditional S2-AI entry points,^2^ the position is often medial to the S1 screw and often requires kyphotic bend of the rod from the S1 to the iliac screw head. Furthermore, there’s extreme medial to lateral angulation of the MIS tulip tabs relative to the lateral to medial angulation of the MIS tabs in the lumbar spine. This makes the rod passage not intuitive.

### 1:34 Key Ideas and Modifications.

The key idea^3^ is to first place the S1 screw to identify the S1 tulip in order to facilitate pelvic screw placement and ease of rod passage. The pelvic screw trajectory can be planned to align with the lumbar rod path based upon the S1 screw position. The key modifications^4,5^ are that the entry point would be more lateral to a traditional S2-AI point, almost at the sacroiliac joint, and the angulation of the tabs would be more vertical given the lateral entry point, and finally, the depth of the screw head can be customized to align with the S1 screw head.

### 2:09 Model Demonstration.

On this model, you can see where the lumbar as well as the S1 screws are generally placed. You can see that in a traditional pelvic fixation at the PSIS, the screws are very prominent and very dorsal. The angulation is also shown. Shown here is the standard S1 entry point. Now shown is the standard S2-AI starting point at the midpoint between the S1 and S2 dorsal foramen. Now we are showing our lateral modification, as well as the traditional pelvic screw. You can see that our modification is lateral to the traditional S2-AI; this will line up nicely with the S1 screw. In this image, you can see the starting point of the traditional pelvic screw depicted in blue, with the S2-AI screw in yellow, our modification in red, and the S1 starting point with the black circle.

### 3:10 Case Example.

This case example is of an 81-year-old gentleman who presented with severe neurogenic claudication. On examination, he was intact with severe radiculopathy. He’s had previous lumbar laminectomies at outside hospitals. His diagnoses are spondylolisthesis, scoliosis, up-down foraminal stenosis at multiple levels after an L3–S1 laminectomy. He’s planned for stage 1, L2–5 oblique lumbar interbody fusion with L5–S1 anterior lumbar interbody fusion, and for stage 2, L2–S1 and pelvis MIS instrumented fusion.

### 3:45 Imaging Review.

Here you can see the sagittal as well as the axial T2-weighted MRIs. Here you can see the parasagittal views demonstrating up-down foraminal stenosis, more specifically at L3–4, L4–5, and L5–S1. You can also see the previous laminectomy defect at L3. This is the CT scan demonstrating the L3 laminectomy defect. AP and lateral x-rays reveal the spondylolisthesis as well as the scoliosis.

### 4:14 Operative Video: Planning.

Here you can see that with navigation, we first plan the trajectory of the pedicle screws. Here with navigation, you can see where we’re planning the S1 pedicle screw. And now you can see the traditional entry point of the S2-AI screw. And now is a lateral modification of that entry point. On the left image, you can see a traditional pelvic screw with our modification on the right.

### 4:45 Pelvic Screw Insertion.

Here you can see using a navigated drill to go through the entry point and through the sacroiliac joint. This is then subsequently tapped using a navigated tap, avoiding the sciatic notch and going through the SI joint. The screw is then subsequently placed and similar to traditional S2-AI screws, this modified screw crosses the SI joint but does not fuse it. This should be taken into consideration when contemplating this method of pelvic fixation. Here you can see the alignment between the S1 screw and the pelvic screw, facilitating ease of rod passage. This is now the contralateral side, starting with a navigated burr almost into the sacroiliac joint itself. This still allows for the medial to lateral angulation to align with the S1 screw. The entry point is subsequently drilled into the depth of the actual burr and marked with navigation, and this is saved. Since the modified S2-AI entry point is close to the SI joint, there is a risk of SI joint widening and skiving. As such, it is critical to use a high-speed burr to create a cortical breach and to advance the instruments slowly with steady axial pressure under navigation. And now you can see the tapping used to further cannulate the screw hole. Here you can see the screw being placed under navigation. You can see that as the screw is buried, it will be lateral to align with the sacral screw, yet medial enough to be ventral to the posterior superior iliac spine.

### 6:16 Rod Placement.

Here you can see what the alignment looks like with the L2–S1 screws with the pelvic screw. Now you can see the rod being passed from cephalad to caudad. With one pass, this can be easily facilitated. Here is another view of the rod being passed from cephalad to caudad on the left side. The lighted sucker can be used to visualize the tulip. Now the rod is being passed on the contralateral side from L2 to S1 and to the pelvis. The set screws are then subsequently placed, and the rod is gently tightened so it does not move.

### 7:17 Postoperative Imaging.

Now you can see intraoperative imaging demonstrating the alignment from the S1 screw to the pelvis. Further intraoperative imaging demonstrates the position of the pelvic screws. Shown here is the pre- and postoperative comparison of the SI joints.

Here are the AP and lateral postoperative radiographs demonstrating the relationship between the screws and rods and the alignment and curvature of the rod in the sagittal plane.

## Disclosures

A. Chan reported personal fees from Alphatec Spine, Carlsmed, and Spineart; and grants from Cervical Spine Research Society outside the submitted work. P. Mummaneni reported personal fees from DePuy/Johnson & Johnson MedTech, Globus, GE/BK Medical, and Medtronic; grants from NREF, ISSG, AO Spine, NIH, PCORI, SLIP II, Pacira, and DoD; stock in Discgenics; and royalties from Springer Publisher and Thieme Publisher outside the submitted work. D. Chou reported personal fees from Globus and Medtronic outside the submitted work.

## Author Contributions

Primary surgeon: Chou. Assistant surgeon: Moghaddamjou, Winans. Editing and drafting the video and abstract: Chou, Moghaddamjou, Yoh, Winans, Chan. Critically revising the work: Chou, Moghaddamjou, Chan, Mummaneni. Reviewed submitted version of the work: Chou, Moghaddamjou, Chan, Mummaneni. Approved the final version of the work on behalf of all authors: Chou. Supervision: Chou.

## Supplemental Information

### Patient Informed Consent

The necessary patient informed consent was obtained in this study.
